# Exploring WNT pathway dysregulation in serrated colorectal cancer for improved diagnostic and therapeutic strategies

**DOI:** 10.3389/fgene.2025.1586867

**Published:** 2025-04-28

**Authors:** Fengzhi Zhu, Helen Hoi Ning Yan, Yin Tong, Yueliang Zhao

**Affiliations:** ^1^ College of Food Science and Technology, Shanghai Ocean University, Shanghai, China; ^2^ School of Public Health, Shanghai Jiao Tong University School of Medicine, Shanghai, China; ^3^ Department of Pathology, School of Clinical Medicine, Queen Mary Hospital, The University of Hong Kong, Pokfulam, Hong Kong SAR, China; ^4^ Centre for Oncology and Immunology, Hong Kong Science Park, Hong Kong SAR, China

**Keywords:** Wnt signaling pathway, serrated colorectal cancer, molecular subtype, tumor microenvironment, precision oncology

## Abstract

**Background:**

Serrated colorectal cancer (SCC) is a rare and aggressive subtype of colorectal cancer. Identifying SCC is crucial due to its high mortality rate and limited therapeutic options. Traditional methods to identify BRAF hotspot mutations and MLH1 methylation are insufficient in clinical practice. This study aims to explore the WNT pathway alterations in the CRC and to develop a WNT-derived subtyping model to identify SCC patients by using multi-OMICs data.

**Methods:**

We included multi-omics data of 1751 colorectal cancer patients from the TCGA and GEO databases, and single-cell transcriptome data of 33 normal and cancer tissues from the SMC study cohort. The comprehensive study process incorporated unsupervised clustering, enrichment analysis, and statistical analysis.

**Results:**

In this study, we investigated WNT pathway alterations in SCC by integrating both bulk and single-cell data into the multi-OMICs framework. The SCC subtype demonstrated significant WNT pathway heterogeneity and a more stable genomic structure. These findings support the development of a WNT-derived subtyping model that accurately identifies SCC patients across different CRC cohorts. In addition, the SCC subtype also presented a distinct immune microenvironment characterized by CD8^+^ T cell exhaustion. Finally, we utilized drug perturbation data to explore the potential drug targets for this severe cancer subtype.

**Conclusion:**

We developed a WNT-derived subtyping method to identify SCC from canonical CRC, which enhances the molecular understanding of this severe cancer subtype and provides potential therapeutic strategies. Our findings suggest that SCC patients may benefit from the HSP90 inhibitor NVP-AUY922, highlighting its potential as a targeted therapy.

## 1 Introduction

Colorectal cancer (CRC) is the third most common and the second most deadly type of cancer worldwide (Sung et al., 2021; Tong et al., 2023). CRC is a heterogeneous oncological disease that develops through two major molecular pathways (Fessler and Medema, 2016; Wang et al., 2021). The conventional adenocarcinoma (CA) pathway is initiated by biallelic inactivation of the APC, and it progresses to cancer through mutations in KRAS and TP53. In contrast, mutations in the APC are uncommon in the serrated colorectal cancer (SCC) pathway, which is initiated by activating mutations in BRAF or KRAS and usually progresses to malignancy through a plethora of epigenetic alterations, microsatellite instability and MLH1 hypermethylation (Fennell et al., 2020; Borowsky et al., 2018; Rustgi, 2013). Due to the molecular homogeneity of CIMP-high, BRAF-mutant CRCs and serrated colorectal cancer (JE et al., 2015), many studies have used CIMP status and BRAF mutation as molecular markers to identify serrated colorectal cancer (Mesteri et al., 2014; Kriegl et al., 2011; Bleijenberg et al., 2022). However, this method has limitations, such as patients with serrated lesions of the CMS4 phenotype showing BRAF wild-type (Fessler and Medema, 2016; Mouillet-Richard et al., 2024).

WNT pathway gene mutations and expression significantly differ between the two oncogenic pathways. In serrated colorectal cancer, WNT pathway gene mutations are common, but most of the APC genes are wild-type and have low activation of the WNT pathway. In contrast, the situation is reversed in conventional adenocarcinomas (Fennell et al., 2020; Borowsky et al., 2018; Murakami et al., 2015).

In this study, using a multi-omics clustering method, we demonstrated that the genomic and transcriptomic components of WNT signaling pathway play crucial roles in driving molecular heterogeneity in CRC. Among these, a unique WNT subtype showed characteristics consistent with SCC, including a high prevalence of BRAF mutations, MLH1 methylation, and microsatellite instability. Furthermore, we found a new set of SCC signatures and validated their predictive accuracy and applicability. Additionally, we comprehensively characterized the clinical, molecular, biological, and immune microenvironment characteristics of serrated colorectal cancer. Moreover, we propose a computational model to predict that patients may benefit from HSP inhibitor NVP-AUY922. Our research aims to enhance biological understanding and guide clinical management.

## 2 Methods

### 2.1 Data source

Multi-OMICs data, including mRNA expression (Illumina HiSeq RNASeqV2), DNA methylation (HM27 and HM450 merge), somatic mutation (MAF files) and somatic copy number variation (CNV GISTIC 2.0), and clinical information of patients with CRC were downloaded from TCGA (PanCancer Atlas) in the cbioprotol database (https://www.cbioportal.org/).

The GEO database (http://www.ncbi.nlm.nih.gov/geo/) was searched for available colorectal cancer datasets for further validation. This study included four independent datasets from the GPL570 platform: GSE38832 (n = 122), GSE39582 (n = 566), GSE17536 (n = 177) and GSE14333 (n = 290). The GSE4045 dataset (serrated colorectal cancer: 8 cases, conventional adenocarcinomas: 29 cases, GPL96 platform), GSE116305 dataset (serrated colorectal cancer: 15 cases, GPL4133 platform), and GSE36758 dataset (serrated colorectal cancer: 11 cases, conventional adenocarcinomas: 15 cases, GPL4133 platform) were also included in the study.

Single-cell transcriptome data (10× genomic sequencing, SMC) were acquired at Synapse (https://www.synapse.org/#!Synapse:syn26844071/) (Joanito et al., 2022).

To compile the annotation table of the WNT pathway genes, we gathered genes from reputable scientific sources, including KEGG (Entry ID: hsa04310) and review articles. After eliminating duplicate genes, a total of 154 WNT genes were included for subsequent analysis (Supplementary Table S1).

Furthermore, 46 colorectal cancer cell lines with gene expression data and 264 drug half-maximal inhibitory concentration (IC50) data were obtained from the GDSC database (https://www.cancerrxgene.org/) for drug sensitivity analysis.

### 2.2 Data preprocessing

TCGA data preprocessing. The expression level of mRNAs was calculated using the log2 (normalised count +1). Select gene promoter region and enhancer region data as DNA methylation levels. All negative values in the DNA methylation and the mRNA expression were considered to be missing values (NA), and using DMwR2 package in the k-nearest neighbor method to fill missing values. For somatic mutation data, after removing synonymous mutations, a binary mutation profile was employed to indicate the presence or absence of a gene mutation. Somatic copy number variant data processed and coded by GISTIC 2.0 were selected (Mermel et al., 2011).

GEO data preprocessing. The gene expression data were normalized independently for each dataset using the affy package. Subsequently, the individual datasets were consolidated into a unified GEO dataset, and batch effects were eliminated through the utilisation of the removeBatchEffect function within the limma package.

Single-cell transcriptome data preprocessing. Low-quality cells were removed if they had less than 200 expressed genes or over 25% unique molecular identifiers (UMIs) from the mitochondrial genome. We removed genes detected in less than 3 cells. Afterwards, all bipartite groups in each sample were removed by using the DoubletFinder package. Batch effects between samples were eliminated using the RunHarmony function, as accessible via the Harmony package. The cell components were annotated using a combination of manual and automated annotation (scHCL package). Single-cell transcriptomic data from 54,593 cells were included in this study. The clustering of diverse cell types was performed using the Seurat package. Data normalization was conducted using the NormalizeData function, with the default scaling parameter of 10,000 and log normalization method. FindVariableGenes function was employed to identify 2,000 genes exhibiting the greatest variance. The data were subsequently standardised using the ScaleData function. Following the principal component analysis of the highly variable genes, the top 30 principal components and a resolution of 0.2 were selected for the subsequent cluster analysis and visual dimensionality reduction by UMAP for dimension reduction. To identify the marker genes of each cluster, the FindAllMarkers and FindMarkers function was employed. The gene expression levels were presented using the FeaturePlot or VlnPlot functions. The clusters were then labelled using known classical marker genes (epithelial cells: KRT18, KRT8 and EPCAM; endothelial cells: PECAM1; B cells: MZB1, JCHAIN and CD79A; T cells: CD3D; myeloid cells: LYZ, FCGR3A and CD68; fibroblasts: DCN, COL1A1, COL1A2 and C1R; mast cells: CPA3) (Zhou et al., 2023; Ma et al., 2023; Pelka et al., 2021; Khaliq et al., 2022). Finally, finer subclusters were further identified by repeating the above operations. The AddModuleScore function was utilized to calculate the SCC gene set score and WNT pathway activity in epithelial cells.

After data preprocessing, 518 CRC patients with matched data of mRNA expression, somatic mutation, CNV and DNA methylation data were selected for subsequent analyses. A total of 1233 CRC patients from GEO datasets were included in this study. scRNA-seq profiles from 54,593 cells were included in this study.

### 2.3 Cluster analysis of WNT gene mRNA expression and somatic mutation using TCGA dataset

Based on the WNT gene mRNA expression and somatic mutation, four widely applied clustering methods (consensus clustering, NMF clustering, K-means clustering with PCA or tSNE dimensionality reduction) were conducted, respectively. The optimal number of clusters was determined through the use of consensus map, average silhouette width and cophenetic scores based on NMF clustering analysis. NMF clustering with 50 iterations of Lee’s method was conducted using the NMF package. The K-means method was employed for consensus clustering in the ConsensusClusterPlus package, with 1,000 replications to ensure the stability and consistency of the clustering results. Since the somatic mutation is binary, we set the binary distance when clustering on the somatic mutation.

Subsequently, integrative clustering analysis was employed to identify CRC subtypes using the iClusterBayes, a Bayesian integrative clustering method designed for the analysis of multi-omics data, implemented in the iClusterPlus package. This involved the analysis of WNT gene mRNA expression and somatic mutation. To ensure consistent and easily reproducible results, MCMC sampling parameters were set (n.burnin = 18,000, n. draw = 12,000). The optimal number of clusters was determined through the use of Bayesian Information Criterion (BIC) and deviation ratio plots between 2 ∼ 9 clusters. The optimal k value was where the curve of BIC and deviation ratio levels off, with the optimal number of clusters determined as k+1.

### 2.4 Identification of signature genes for CRC subtypes

The limma package was employed to perform differential expression analyses between tumour samples and normal samples, as well as each subtype and other subtypes. This was done to identify the signature genes of each subtype in the TCGA dataset. The following criteria were employed to identify the signature genes: (i) A comparison was conducted between each subtype and other tumour samples (all P < 0.05 and log2|fold change| > 1). Due to the numerous upregulated genes in W3 and W4, numerous downregulated genes in W2, we adjusted the criteria for the three subtypes (P < 0.05, W2: log2|fold change| > 0.3; W3: log2|fold change| > 1.4; W4: log2|fold change| > 1.1). (ii) A comparison was conducted between tumour samples and normal samples (all P < 0.05 and log2|fold change| > 2). (iii) The signature genes were unique in the CRC subtypes.

The pROC package was also used for calculating the multi-class area under curve (AUC) to ensure the geneset’s plausibility.

### 2.5 Enrichment analysis

518 TCGA tumor samples were identified by CMS classification (CMS1 = 89, CMS2 = 154, CMS3 = 87, CMS4 = 181, NA = 81) using the CMScaller package (Eide et al., 2017). The WNT signaling pathway gene set (Supplementary Table S1) was enriched by the GSVA package, and the result of the enrichment was used as the activity of the WNT pathway. We used the same method to calculate the GSVA scores of the existing serrated carcinoma-associated gene sets (Joanito et al., 2022; Laiho et al., 2007; Chen et al., 2021) and the SCC signatures in this study (Supplementary Table S2). Biological process enrichment analysis was performed by the clusterProfiler package, and the annotated gene sets were obtained from the MSigDB database (https://www.gsea-msigdb.org/gsea/msigdb/).

### 2.6 Molecular subtyping validation based on GEO dataset

The nearest template prediction (NTP, Gene Pattern) algorithm (Hoshida, 2010) was employed to classify CRC patients in the GEO datasets via the subtype signature genes from the TCGA dataset. Furthermore, subclass mapping analysis (SubMap, Gene Pattern) (Hoshida et al., 2007), an algorithm for assessing the similarity of molecular subtypes between independent datasets based on gene expression profiles, was employed to ascertain whether the subtypes identified in the TCGA and GEO datasets presented a significant degree of similarity.

### 2.7 Copy number variation estimation

The InferCNV package was employed to ascertain the copy number variations (CNVs) in epithelial cells, with reference to transcriptome profiles (inferCNV of the Trinity CTAT Project, https://github.com/broadinstitute/inferCNV). The Epithelial cells in normal samples were used as reference cells for CNV estimation. The copy number variation status of each epithelial cell was mapped between −1 and one for normalization and then the sum of squares was calculated as the copy number variation value to characterize the genomic stability of the epithelial cells.

### 2.8 Immune infiltration analysis

To understand the immune infiltration of colorectal cancer subtypes, we performed immune infiltration analysis using RNA-Seq expression matrix to predict the relative abundance of immune cell infiltration by using the xCell package (Aran et al., 2017).

### 2.9 Estimation of drug response in clinical samples

Predicting responsiveness to immune checkpoint inhibitors (ICBs) in CRC patients using TIDE (Jiang et al., 2018) (http://tide.dfci.harvard.edu/). Using the oncoPredict package, drug (chemotherapy or targeted drug) IC50, were predicted for CRC patients (Iorio et al., 2016; Maeser et al., 2021).

### 2.10 Connectivity map analysis

Connectivity map (CMap) analysis was conducted to ascertain the potential therapeutic efficacy of drug candidates in SCC. The initial step was to conduct a differential expression analysis on tumour and normal samples. Subsequently, the 300 genes exhibiting the most pronounced fold change (150 upregulated genes and 150 downregulated genes, P < 0.05) were submitted to the CMap website (https://clue.io/query) (Lamb et al., 2006; Subramanian et al., 2017).

### 2.11 Statistical analysis

All statistical analyses were performed using the R software (version 4.2.1). The associations between the CRC subtypes or SCC signatures scores high and low groups (separated by median) and survival were assessed by Kaplan–Meier survival analyses. The categorical data were analyzed by chi-square test or Fisher exact test. Differences in continuous data between multiple groups were determined by Kruskal–Wallis rank sum test. A two-tailed P value < 0.05 was regarded as statistical significance (ns: P = not significant; *: P < 0.05; **: P < 0.01; ***: P < 0.001; **** *: P < 0.0001).

## 3 Results

### 3.1 WNT pathway genes characterize CRC patients into five unique subtypes

**FIGURE 1:**
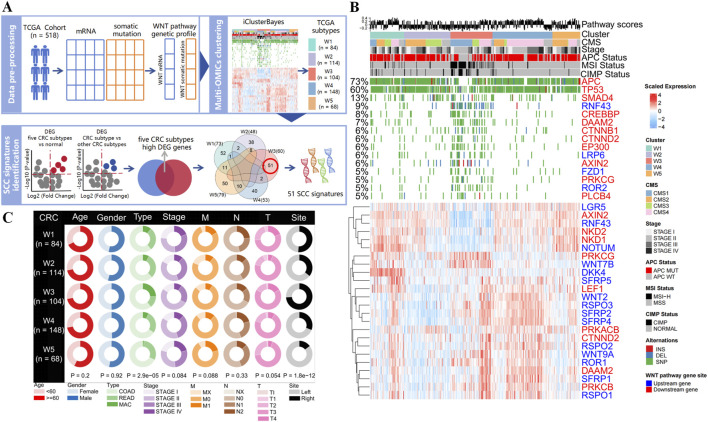
Identification of CRC subtypes using WNT pathway gene expression profiles and mutation profiles. **(A)** Overview of methods for developing WNT-derived subtyping and identifying SCC signatures. **(B)** Heatmaps show gene expression and mutation patterns of the CRC subtypes identified by integrative clustering analysis for WNT pathway. The distribution of WNT pathway score, CMS phenotype, cancer stage, APC status, microsatellite stability status, and CpG island methylation status in each subtype is shown (all features posterior probability >0.9). **(C)** Circle plot of clinical features of CRC subtypes in the TCGA dataset (COAD: colon adenocarcinoma; READ: rectum adenocarcinoma; MAC: mucinous adenocarcinoma of the colon and rectum).

To answer the question of whether the CRC heterogeneity is shaped from WNT pathway alterations, we conducted a multi-OMICs analysis of CRC patients (n = 518) from TCGA database, including 154 WNT pathway genes (63 upstream genes and 91 downstream genes, Figure 1A; Supplementary Table S1). On this dataset, cluster rank for the WNT-pathway gene expression (WNT-mRNA) and the WNT-pathway gene mutations (WNT-mutation) were first computed to guide the patient clustering (WNT-mRNA cluster rank: 3; WNT-mutation cluster rank: 2, Supplementary Figures S1,S2). The results of independently clustering by WNT-mRNA and WNT-mutation were significantly overlapped with each other (all P < 0.001, Supplementary Figure S3A; Supplementary Table S3). This finding suggests a strong association between the genomic and transcriptomic dysregulation of the WNT signaling pathway components in defining molecular subtypes of CRC (NMF clustering, P < 0.001; Consensus clustering, P = 0.1789; K-means clustering with PCA or tSNE dimensionality reduction, p < 0.01, Supplementary Figure S3B; Supplementary Table S3).

Subsequently, we conducted the cross-clustering analysis by both WNT-mRNA and WNT-mutation data to identify the molecular clusters of CRC. The BIC and deviance ratio plots showed that five clusters (W1, n = 84; W2, n = 114; W3, n = 104; W4, n = 148; W5, n = 68) stood for the optimal solution (Figure 1B; Supplementary Figures S4A,S5A). More biological values were added by the chi-square test that revealed the consistency between the five integrative clusters and the stand-alone clustering results (all P < 0.001, Supplementary Figure S5C; Supplementary Table S3), further demonstrating the contribution of WNT signaling pathway to the molecular heterogeneities of CRC patients. Moreover, we found that mutation in WNT downstream genes and differential expression of WNT upstream genes were the major factors driving the CRC clustering (all posterior probabilities >0.9), indicating the WNT pathway activities are regulated mainly by upstream transcriptome regulations and downstream gene mutations (Figure 1B; Supplementary Figures S4B,S5A). Notably, the APC gene is the key suppressor gene in the WNT pathway, and its normal activation inhibits the pathway activation (6). We detected the highest mutation frequency (73%) in the APC gene among the five cancer clusters, but the APC wild-type was significantly enriched in W3 cluster (P < 0.001). Meanwhile, mutations in WNT pathway genes are prevalent in W3 cluster, which often occurs during the transformation of serrated colorectal polyps to malignant tumors (Fennell et al., 2020; Borowsky et al., 2018).

In addition, we compared the pathological characteristics and survival prognostic status of five CRC clusters. We found that the W3 cluster was significantly associated with tumor type and colon site (all P < 0.001, Figure 1C). Mucinous adenocarcinoma is mainly concentrated in the W3 cluster, which is more likely to be detected in the right colon. Kaplan-Meier survival analysis revealed that among the five subtypes, W3 cluster had the worst prognosis in terms of OS and DSS in the first 50 months, whereas better prognosis in terms of DFS and PFS were observed (Supplementary Figure S5B). These results demonstrated that the heterogeneities in the WNT signaling genes have reshaped a W3 subtype with distinct molecular alterations, setting it apart from other subtypes in many clinical aspects.

### 3.2 Identify the serrated colorectal cancer subtype by the WNT cluster 3

**FIGURE 2:**
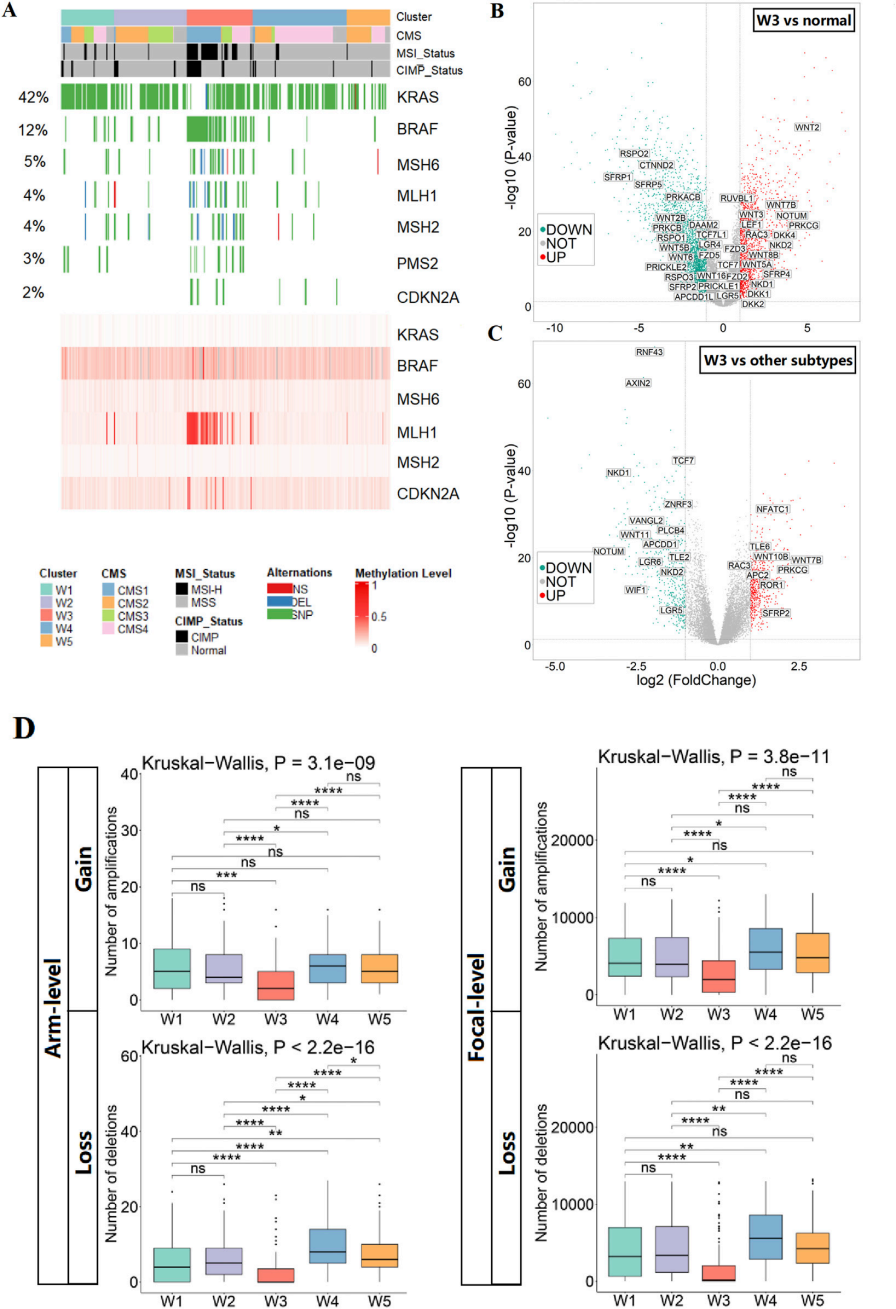
Identification and characterization of SCC subtypes. **(A)** Heatmap show the status of SCC key molecular features in CRC subtypes (mutation of genes in the upper panel; methylation of genes in the lower panel). **(B)** Volcano plot of differentially expressed genes of the WNT pathway in SCC subtypes versus normal tissues. **(C)** Volcano plot of differentially expressed genes of the WNT pathway in SCC subtypes versus other subtypes. **(D)** The CNV burden of gains and losses, at both arm and focal, in the five CRC subtypes.

The molecular contribution of CIMP-H and BRAF-mutant to APC mutant-free cancer initiation of serrated type CRC has been widely discussed (JE et al., 2015). Strikingly, we found that the BRAF mutant was significantly enriched in APC-wild-type W3, accompanied by significant enrichment of CIMP-H (all P < 0.001, Figure 2A). In addition, mutations of mismatch repair proteins (MSH6, MLH1, MSH2, and PMS2) and methylation of CDKN2A were coordinated in the same cluster (Figure 2A). These findings revealed the robust characteristics of serrated lesions found in W3 cluster. Notably, the W3 cluster exhibited the BRAF-mutated CMS1 phenotype and the KRAS-mutated CMS4 phenotype, but the CMS2 phenotype was absent (Figure 2A). Previous study has shown that BRAF-mutated CMS1 phenotype and the KRAS-mutated CMS4 phenotype are the predominant phenotypes in SCC, whereas the CMS2 phenotype is almost exclusively present in CA, exhibiting activation of the WNT pathway (Fessler and Medema, 2016). These findings suggest that our approach is corroborated by CMS studies and compensates for the earlier shortcomings of identifying serrated lesions based on independent molecular features (CIMP-H or BRAF mutation). In addition, we calculated several SCC signature scores by using the SCC differential expressed genes from published studies (Joanito et al., 2022; Laiho et al., 2007; Chen et al., 2021), which all were significantly higher in W3 compared with the other clusters (all P < 0.001, Supplementary Figure S6; Supplementary Table S2). All the results proved the successful classification of the SCC subtype from CRC using our WNT cluster 3 (W3) model.

To investigate alterations in the WNT pathway in SCC subtypes, we evaluated WNT pathway activity, focusing on alterations in key WNT pathway genes. We found that the WNT pathway was activated in the SCC subtype relative to normal tissues, as evidenced by the upregulation of WNT ligands (WNT2 and WNT3) and the transcription factor LEF1, and the downregulation of repressor genes (SFRP1 and SFRP5). However, compared with other subtypes, the WNT pathway was relatively repressed in the SCC subtype, which showed upregulation of repressor genes APC2 and downregulation of transcription factor TCF7 (Figures 2B,C; Supplementary Figure S7). The above results suggest that the WNT pathway activities is higher in SCC than the normal tissue, but lower than the other CRC samples. To characterize the genomic alterations of SCC subtypes, a comparison of somatic copy number variations was conducted among the five subtypes. Overall, the number of chromosomal aberrations in the SCC subtype was significantly lower than other subtypes at both the arm and focal levels, suggesting that the genomic structure of the SCC subtype is stable (Figure 2D).

### 3.3 W3 signature identifies SCC cases from independent CRC cohorts

**FIGURE 3:**
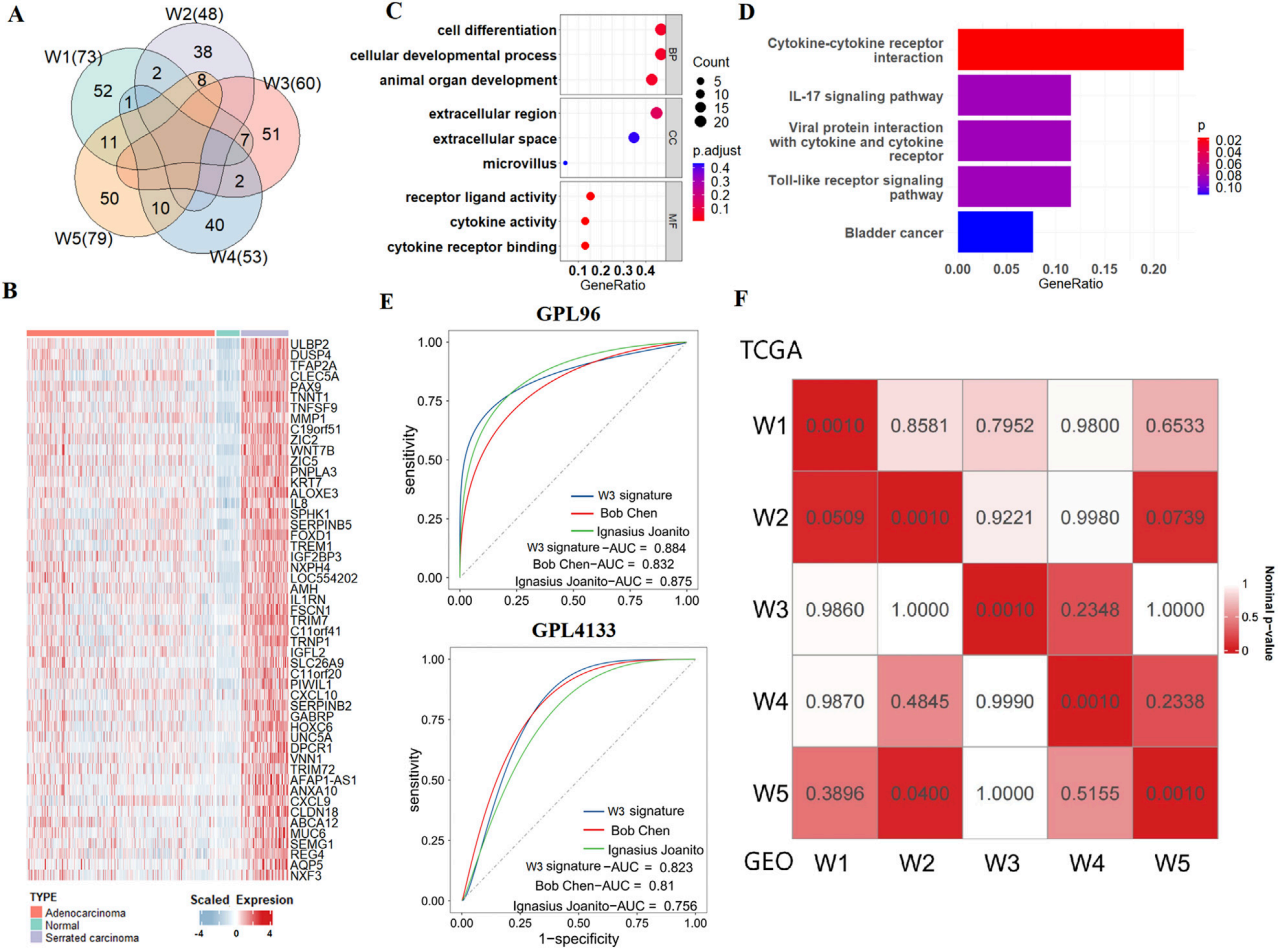
SCC subtype signatures and validation of molecular typing. **(A)** Venn diagram shows the numbers of unique and overlapping differentially expressed upregulated genes when comparing each subtype to other tumour samples as well as each subtype to adjacent normal samples. **(B)** Heatmap of SCC signatures in normal tissues, CA, and SCC. **(C)** Bubble plot of GO enrichment for SCC signatures. **(D)** Bubble plot of KEGG enrichment for SCC signatures. **(E)** ROC curves show the AUC values of the SCC signatures gene set and other serrated-related gene sets in GEO verification sets. **(F)** SubMap analysis show similarity between 5 WNT subtypes in TCGA and GEO datasets.

According to the OMICs analysis of the five WNT clusters, we inferred that W3 corresponds to the serrated colorectal cancer (SCC) subtype, while the others correspond to the conventional adenocarcinoma (CA). To investigate the unique biological processes of the SCC subtype, a supervised analysis (see the “Methods” and Figure 1A) highlighted 231 WNT signature genes, distributed as follows: 52 genes for W1, 38 genes for W2, 51 genes for W3, 40 genes for W4 and 50 genes for W5 (Supplementary Figure S8; Figures 3A,B; Supplementary Table S4). Functional enrichment analysis of the 51 SCC-specific genes using KEGG and GO terms revealed their association with immune activation, cellular interactions and cell differentiation (Figures 3C,D). These findings are consistent with the biological features of serrated polyps that originate from colon-to-gastric metaplasia (Chen et al., 2021).

To assess the ability of our SCC gene signatures to accurately identify the SCC subtype in cancer patients, we tested it across independent CRC cohorts that collected from GEO database. We used three datasets for this purpose: GSE4045 (SCC: 8 cases, CA: 29 cases, GPL96 platform), GSE116305 (SCC: 15 cases, GPL4133 platform), and GSE36758 (SCC: 11 cases, CA: 15 cases, GPL4133 platform). We integrated GSE116305 and GSE36758 together and named it as GPL4133 dataset. The results show that by using our W3 SCC signature, the SCC cases can be successfully distinguished from the conventional adenocarcinoma (CA) cases in all the three testing cohorts. We then compared the prediction accuracy of our W3 gene signatures with those from two other studies Joanito et al. (2022) and Chen et al. (2021), as detailed in Supplementary Table S2). Our W3 signatures achieved AUC values of 0.924 in the TCGA training set, and 0.884 and 0.823 in the GEO validation sets, which were higher compared to the other SCC gene sets (Supplementary Figure S9; Figure 3E). These results suggest that our SCC gene signatures are effective at identifying SCC, providing a more accurate assessment of the risk associated with this subtype.

To verify the robustness of the typing results, we further employed NTP algorithm to predict the WNT subtypes of patients from the integrated CRC dataset comprising four independent cohorts, including GSE38832, GSE39582, GSE17536 and GSE14333. The strong correlation between the GEO and TCGA dataset subtypes, as demonstrated by SubMap analysis, validates the existence of the W3 and the other 4 WNT subtypes across independent cohorts and underscores the generalizability of our study (Figure 3F).

### 3.4 Identification of SCC single cells from scRNA datasets

To explore single-cell transcriptional alterations in CRC, we generated scRNA-seq profiles from 10 normal samples and 23 colorectal cancer samples (6 cases APC wild-type) using 10× genome sequencing data (Supplementary Figure S10). In this study, we identified eight major epithelial cell clusters in colorectal cancer, after re-clustering of 14,454 epithelial cells (Supplementary Figure S11A). To distinguish tumor epithelial cells from normal epithelial cells, we examined the difference in epithelial cell distribution between tumor and normal samples, and found that cells of clusters 0, 1, and 4 were almost exclusively present in the tumor tissues. Combined with the feature plot of markers for each epithelial cell sub-cluster, further confirmed that the cells in clusters 0, 1, and 4 were tumor epithelial cell clusters (Supplementary Figures S11A–C).

**FIGURE 4:**
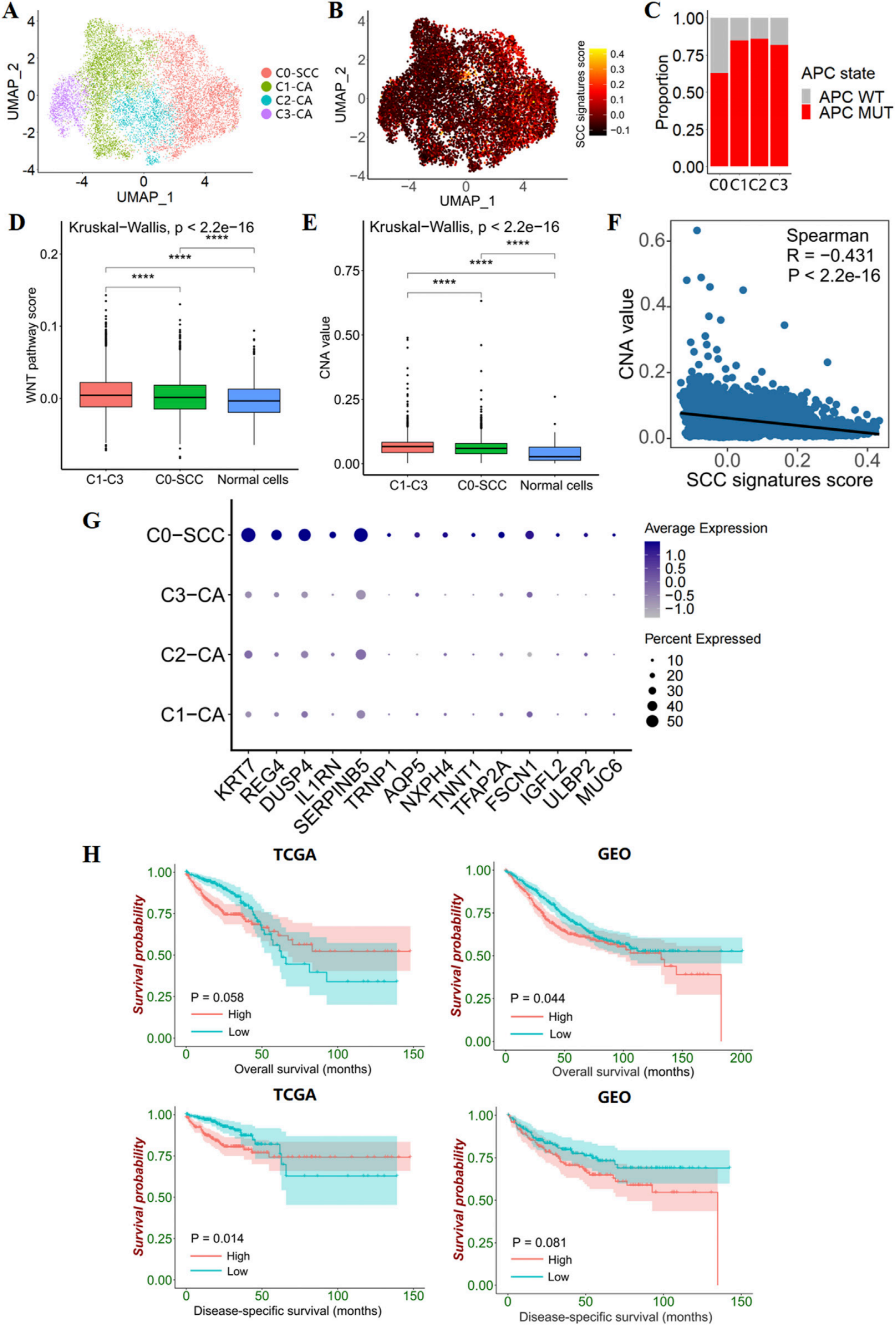
Epithelial cell subclusters in CRC. **(A)** UMAP plot show 4 clusters for 11,695 tumor epithelial cells. **(B)** Feature plots present the SCC signatures AddModuleScore for each tumor epithelial cells. **(C)** Bar plots show the proportion of APC-mutant patients versus APC wild-type patients in the 4 tumor epithelial clusters. **(D)** Box line plot show the differences in WNT pathway activity between CA, SCC and transit amplifying cells. **(E)** Box line plot show differences in genomic structural stability between CA, SCC and transit amplifying cells. **(F)** The correlation between CNV value and SCC signatures AddModuleScore. **(G)** Dotplot plots illustrate 14 differentially expressed SCC signatures in the 4 tumor epithelial clusters. **(H)** Kaplan–Meier survival curves show survival differences between groups with high and low GSVA scores (grouping by median scores) for the 14 differentially expressed SCC signatures.

To further investigate the heterogeneity of different tumor epithelial cells, we re-clustered the tumor epithelial cells to generate four subclusters (Figure 4A). Among the four subclusters, the tumor epithelial cells from the APC wild-type samples were mainly in the 0 cluster, which presented high SCC signature scores (Figures 4B,C). Therefore, we defined the 0 cluster as an SCC epithelial cell cluster, and the remaining clusters as CA epithelial cell clusters. To investigate the WNT pathway activity and genomic stability in these two types of tumor epithelial cells, the WNT pathway AddModuleScore and copy number alteration (CNA) score were calculated. It was found that the WNT pathway AddModuleScore and CNA score of both types of tumor epithelial cells were significantly higher than normal epithelial cells, and the WNT pathway AddModuleScore and CNA score of CA epithelial cells were significantly higher than SCC epithelial cells, indicating the existence of WNT pathway activation in SCC, but the degree of activation was limited; At the same time, SCC epithelial cells have malignant potential, but the genomic structure is relatively more stable, which corresponds with previous results (Figure 2D; Figures 4D,E; Supplementary Figure S7,S12).

In addition, SCC signatures were negatively correlated with the CNA value in CRC tumor epithelial cells, suggesting that SCC signatures have value in predicting genomic structural stability (Figure 4F). Specifically, we found that 14 out of 51 SCC signatures were specifically overexpressed in SCC epithelial cells, and the GSVA score of the 14 SCC single-cell signature gene set was correlated with patient survival (Figures 4G,H). Higher scores predict poorer survival, indicating that the 14 SCC single-cell signature gene set has prognostic value.

### 3.5 Serrated colorectal cancer present T cell exhaustion and immunosuppressive tumor microenvironment

**FIGURE 5:**
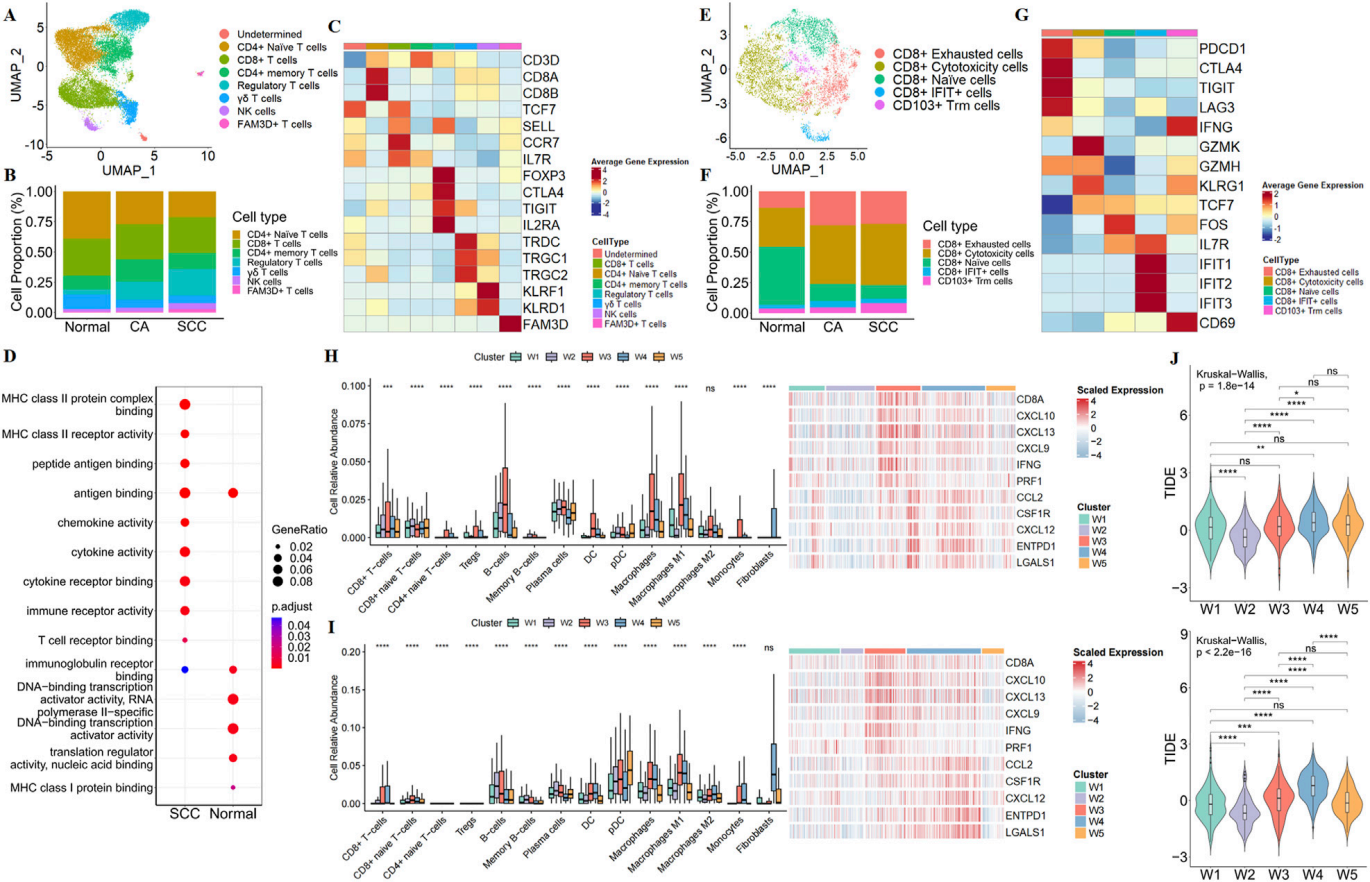
Characterization of the immune microenvironment in SCC. **(A)** UMAP plot show the association of T cell clusters with each cell type defined. **(B)** Bar plot show the percentage of each type of T cells in normal tissue, CA and SCC. **(C)** Heatmap show illustrated canonical markers of each T cells. **(D)** GO enriched bubble plots show the differences in the biological processes of CD8^+^ T cells in normal tissue and SCC. **(E)** UMAP plot show the association of CD8^+^ T cell clusters with each cell type defined. **(F)** Bar plot show the percentage of each type of CD8^+^ T cells in normal tissue, CA and SCC. **(G)** Heatmap showe illustrated canonical markers of naïve, exhaustion, cytotoxicity function in CD8^+^ T cell. Relative abundance of immune-related cells, and immune-related gene expression maps for CRC subtypes in the TCGA **(H)** and GEO **(I)**. **(J)** TIDE scores of CRC subtypes in TCGA (up) and GEO (down).

Next, clustering and UMAP visualization were performed on the T-cell components (Figure 5A). Based on known markers, 7 major T cell types were identified (Figure 5C). Compared to normal and CA samples, we found a reduced proportion of CD4^+^ naive T cells and an improved proportion of regulatory T cells (Tregs) in SCC samples (Figure 5B). To confirm these findings, we performed immune infiltration analysis on the TCGA and GEO datasets. This analysis revealed that the SCC subtypes showed immuno-rejective properties, including the relative predominance of regulatory T cells (Tregs) and M2 macrophages, as well as the activation of immunosuppressive genes (CCL2, CSF1R, CXCL12, ENTPD1, and LGALS1) (Figures 5H,I; Supplementary Figure S13). These results suggest that SCC has a broader immunosuppressive tumour microenvironment.

Due to CD8^+^ T cells are recognized to play a core role in anti-tumor immunity, our research focused on alterations in CD8^+^ T cells. We re-clustered CD8^+^ T cells, and found that although the proportion of CD8^+^ T cells was similar in normal samples and SCC samples, GO analysis showed that the function of CD8^+^ T cells was altered in SCC (Figure 5D). Meanwhile, a large number of CD8^+^ naive T cells were converted to CD8^+^ cytotoxicity T cells and CD8^+^ exhausted T cells, suggesting that the cytotoxicity produced by CD8^+^ T cells to exert anti-tumor immunity was accompanied by exhaust (Figures 5E–G). The above results imply that the SCC may be insensitive to ICBs. We then employed TIDE algorithm to evaluate the potential responsiveness of the SCC subtype to ICBs. The results revealed that the SCC subtype had the second highest degree of tumor immune dysfunction and exclusion score (TIDE score) after the W4 subtype (Figure 5J). This suggests that the SCC subtype has a high immune escape ability and a poor response to immune checkpoint inhibition therapy.

### 3.6 HSP is the potential therapeutic target for SCC

**FIGURE 6:**
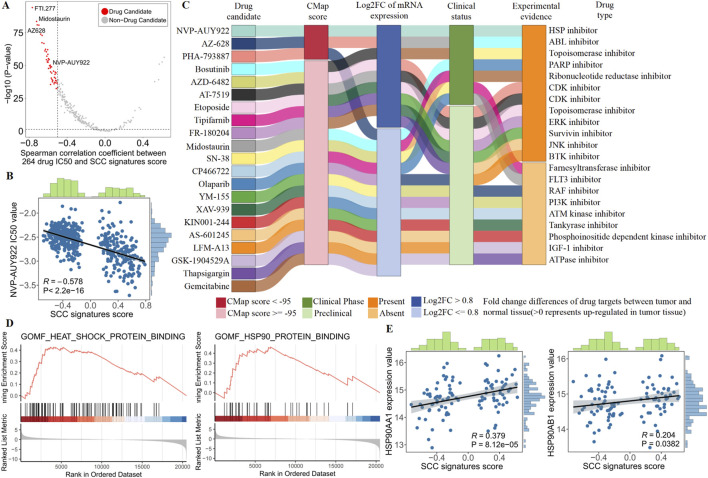
Identify the most promising therapies agents for SCC patients based on multi-source evidence. **(A)** Scatter plot showing the Spearman correlation between 264 drug IC50 and SCC signatures score (red dots represent drug candidate). **(B)** The spearman correlation between SCC signatures scores and NVP-AUY922 IC50 value in TCGA patients. **(C)** Sankey plot displays CMap results for 21 out of 48 candidate drugs. **(D)** GSEA enrichment of heat shock protein (HSP) in SCC patients. **(E)** The spearman correlation between SCC signatures scores and NVP-AUY922 targets (HSP90AA1 and HSP90AB1) expression value in SCC patients.

To identify candidate drugs with higher sensitivity for SCC subtypes, we first calculated the Spearman correlation between the IC50 values of 264 targeted drugs and SCC signature scores in the TCGA dataset. From this analysis, we selected 48 candidate drugs that had a correlation coefficient of less than −0.5 and a correlation P-value of less than 0.05 (Figure 6A; Supplementary Tables S5,S6). Among them, we found a significant negative correlation between the IC50 value of the HSP inhibitor NVP-AUY922 and SCC signatures scores, suggesting that inhibiting the HSP pathway may be crucial for treating SCC patients (Figure 6B).

Although SCC subtypes showed a higher drug sensitivity to NVP-AUY922, above analyses alone could not support the conclusion that HSP inhibitor had therapeutic effects in SCC. Therefore, we conducted multiple perspective analyses to further evaluate the potential of HSP inhibitor for treating SCC. First, Connectivity MAP (CMap) analysis indicated that NVP-AUY922 (HSP inhibitor) had the highest CMap score ranking (−98.59), suggesting that the gene expression pattern of NVP-AUY922 was opposite to the SCC-specific expression pattern (Supplementary Tables S7,S8). In other words, NVP-AUY922 can reverse the SCC-specific gene expression pattern to the normal tissue level, indicating its potential therapeutic effect on SCC. Second, differential expression analysis revealed abnormally high expression of NVP-AUY922 targets (HSP90AA1 and HSP90AB1) in SCC, suggesting that inhibition of NVP-AUY922 targets could lead to significant therapeutic effects. Third, we performed comprehensive literature search using PubMed to find the experimental and clinical evidence supporting the use of NVP-AUY922 for treating CRC, further indicating its potential clinical application for SCC (Figure 6C; Supplementary Table S8). Collectively, robust *in vitro* and *in silico* evidence suggests that targeting the HSP pathway is key to treating SCC. Specifically, NVP-AUY922, which acts on HSP targets, emerges as the most promising drug for treating SCC patients due to its ability to inhibit the HSP pathway effectively.

In order to obtain more biological support, we conducted biological process enrichment in SCC patients and examined heat shock protein (HSP) pathway targets. We found SCC patients were strongly enriched in heat shock proteins, and SCC signature scores were significantly positively correlated with HSP pathway targets (HSP90AA1 and HSP90AB1, Figures 6D,E). These results revealed the presence of abnormal heat shock proteins in SCC patients, highlighting the potential of NVP-AUYH22 in treating SCC by inhibiting the HSP pathway.

## 4 Discussion

SCC is a unique subtype of colorectal cancer, and robust identification of SCC will play an essential role in accelerating the application of precision oncology (Roerink et al., 2018). Distinct from previous independent molecular feature-based classifications (Mesteri et al., 2014; Kriegl et al., 2011; Bleijenberg et al., 2022), molecular subtypes focusing on WNT pathway alterations compensate for the shortcomings of previous methods and promote our understanding of the WNT pathway mechanism underlying CRC. Herein, this study revealed that WNT signaling pathway components at the transcriptional and mutation levels play a crucial role in driving molecular heterogeneity in CRC. Next, we identified and characterized subtypes of SCC in the TCGA dataset, summarized multi-omics SCC gene signatures, and validated them in independent GEO datasets, clearly demonstrating the robustness of identifying SCC subtype through WNT pathway alterations. The clinical characteristics, genomic characteristics, tumour immune microenvironment and potential chemotherapeutic agents of the SCC subtype were further explored, and the SCC subtypes showed extreme heterogeneity.

The SCC subtype identified by the WNT pathway alteration was significantly enriched in BRAF mutations, MLH1 methylation, microsatellite instability, highly mutated mismatch repair protein genes and APC wild-type, accompanied by low activity of the WNT pathway and relatively high stability of the genome structure, which are SCC features consistent with previous studies (Fennell et al., 2020; Borowsky et al., 2018). Despite the low activation of the WNT pathway in the SCC subtype, some aberrant activation still exists, which results from differential downregulation expression of the upstream suppressor genes (RNF43 and ZNRF3) in the WNT pathway (Bond et al., 2016). In addition, we detected prevalent mutations in genes downstream of the WNT pathway in the SCC subtype, which is considered a hallmark of the transformation of serrated polyps to malignant tumors (Fennell et al., 2020; Borowsky et al., 2018). Our study emphasizes the critical role of WNT pathway upstream gene expression and downstream gene mutations in SCC progression.

The tumor immune microenvironment is closely related to the occurrence, progression, and treatment of cancer (Li et al., 2021; Li et al., 2024). Therefore, we analyzed the immune microenvironment of patients with SCC at the bulk and single cell level, and found that an exhaust of CD8+T cells and a more generalized immunosuppressive tumour microenvironment in SCC despite some immune activation. This finding was also corroborated in a single-cell study, which demonstrated that although the microenvironment of serrated carcinoma precursors is generally immune-activated, some immunosuppressive cells (anti-inflammatory macrophages, regulatory T cells, and fibroblasts, among others) are also present in the early stages of tumors and further accumulate (Zhou et al., 2023). Although immunoinflammatory properties contribute to anti-tumor immunotherapy, the extensive immunosuppressive tumour microenvironment limits the efficacy of immunotherapy in SCC patients (Rooney et al., 2015; McGranahan et al., 2016; Mariathasan et al., 2018).

We found that SCC patients tended to receive help from NVP-AUY922. NVP-AUY922, a heat shock protein 90 (HSP90) inhibitor, enhances TRAIL induced apoptosis in colorectal cancer cells by inhibiting the JAK2-STAT3 Mcl-1 signaling pathway, and can also enhance the cytotoxic effects of various chemotherapy drugs in CRC (Lee et al., 2015; Lee et al., 2017). Earlier studies have revealed that the lack of sustained inhibition of HSP90 is key to anti-BRAF therapy insensitivity in BRAF-mutant tumours (Eroglu et al., 2024; Paraiso et al., 2012). Consequently, SCC patients may be potential candidates for evaluating the treatment efficacy of a combination of BRAF and HSP90 inhibitors. While further studies and validation experiments are needed, related drugs hold promise for developing targeted therapies to treat SCC. In addition, multiple experiments found that NVP-AUY922 played a critical role in various cancer treatments. NVP-AUY922 inhibited the activity of non-small cell lung cancer, and could enhance the sensitivity of clear cell renal cell carcinoma to sunitinib (He et al., 2022; Chen et al., 2024). A phase I clinical trial showed that NVP-AUY922 can effectively inhibit tumor growth, angiogenesis, and metastasis in glioblastoma, melanoma, and glioblastoma (Eccles et al., 2008). These evidences highlight the enormous clinical potential of NVP-AUY922 in the treatment of SCC.

Admittedly, this study has several limitations that need to be acknowledged. First, the 5 WNT subtypes were potentially biased, as we focused exclusively on WNT pathway gene expression and mutation profiles, without integrating other omics data that could influence pathway alterations in CRC, such as CNVs and DNA methylation. To address this bias, it is necessary to integrate other omics data, to develop a more comprehensive SCC identification system. Second, incomplete data cannot directly use our WNT-derived model. As in this study, the WNT classification model is not directly applied on the CRC cohorts collected from GEO since they do not have mutation data, so we used the expression signature as an alternative approach for testing. We expect that the testing dataset with both gene mutation and expression data should achieve an even better SCC classification by applying our full WNT model. Additionally, studying SCC presents significant challenges. Currently, there are no commercially available human SCC cell lines for research, and there is a severe shortage of tumor models, thus hindering experimental validation of candidate drugs. The advantage of this study is the initial screening of candidate drugs with great clinical translational potential. In future research, it is expected to validate candidate drugs through cell and animal experiments, ultimately achieving clinical application, which will help improve the treatment strategy for SCC.

## 5 Conclusion

In summary, we propose a robust and reliable clustering recognition system for SCC via in-depth analysis of the WNT pathway, which has considerable potential for applications in CRC and will promote precision oncology.

## Data Availability

The original contributions presented in the study are included in the article/Supplementary Material, further inquiries can be directed to the corresponding authors.
