# Supplementary material for: Exploring WNT pathway dysregulation in serrated colorectal cancer for improved diagnostic and therapeutic strategies
Source: Front Genet. 2025 Apr 28;16:1586867. doi: 10.3389/fgene.2025.1586867 (PMC12066562; doi:10.3389/fgene.2025.1586867)
Supplement: Supplementary file 2 [file Table1.docx]

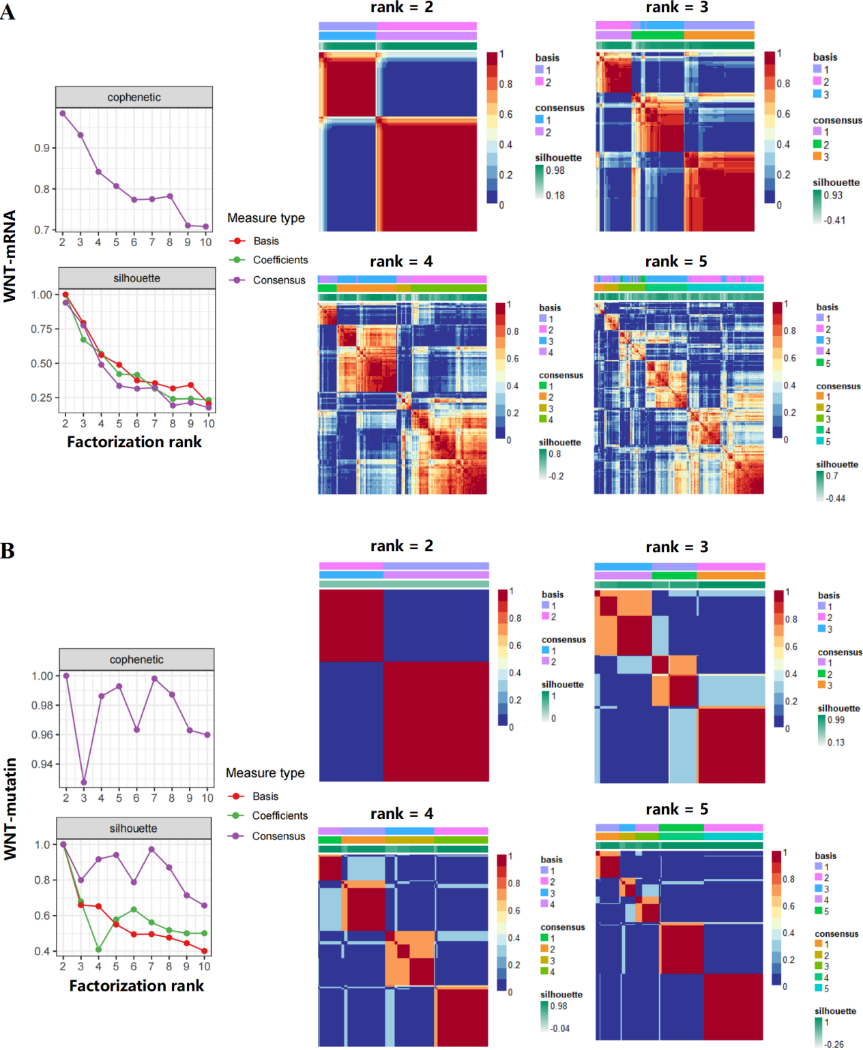


**Figure S1** NMF clustering determines the optimal number of clusters for WNT pathway gene expression profile **(A)** and mutation profile **(B)**.


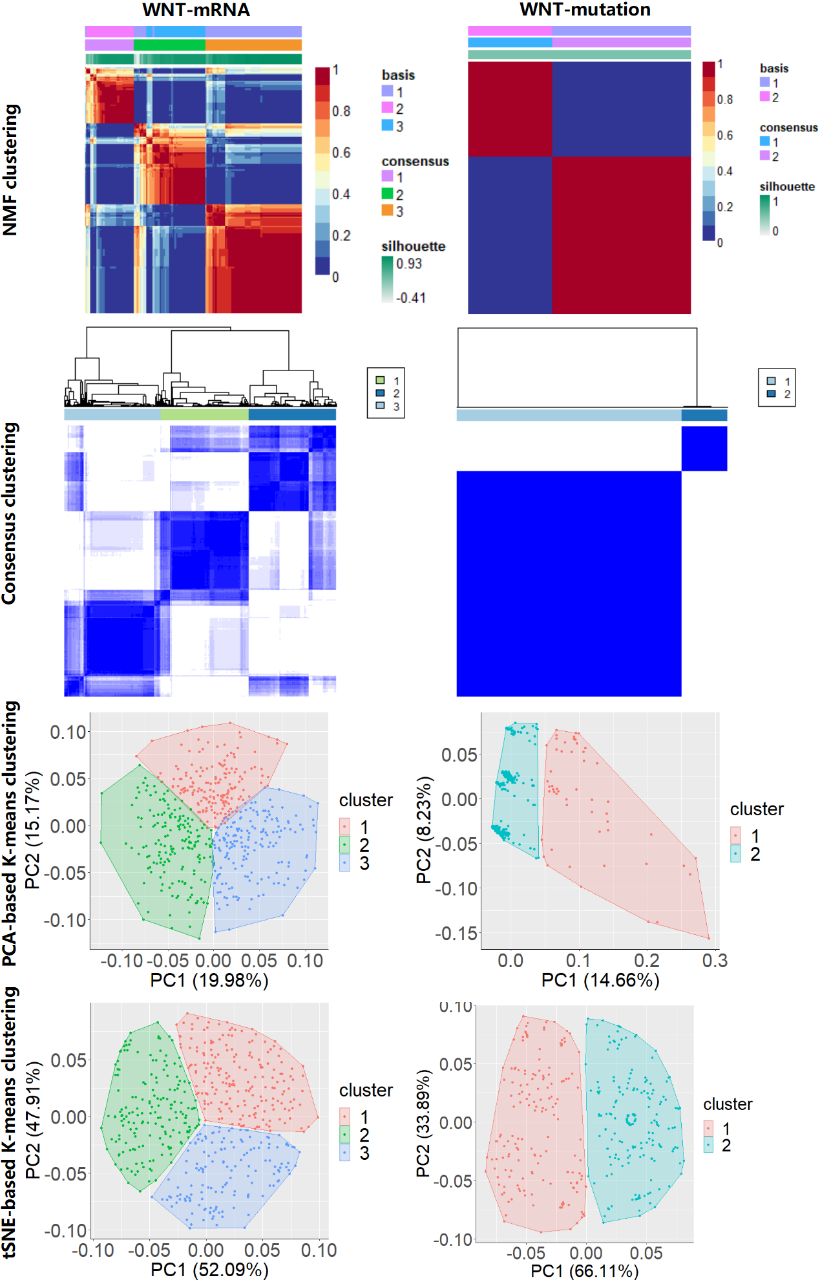


**Figure S2** Consensus clustering, NMF clustering and K-means clustering with PCA or t-SNE clustering diagrams of WNT pathway gene expression and mutation profiles.


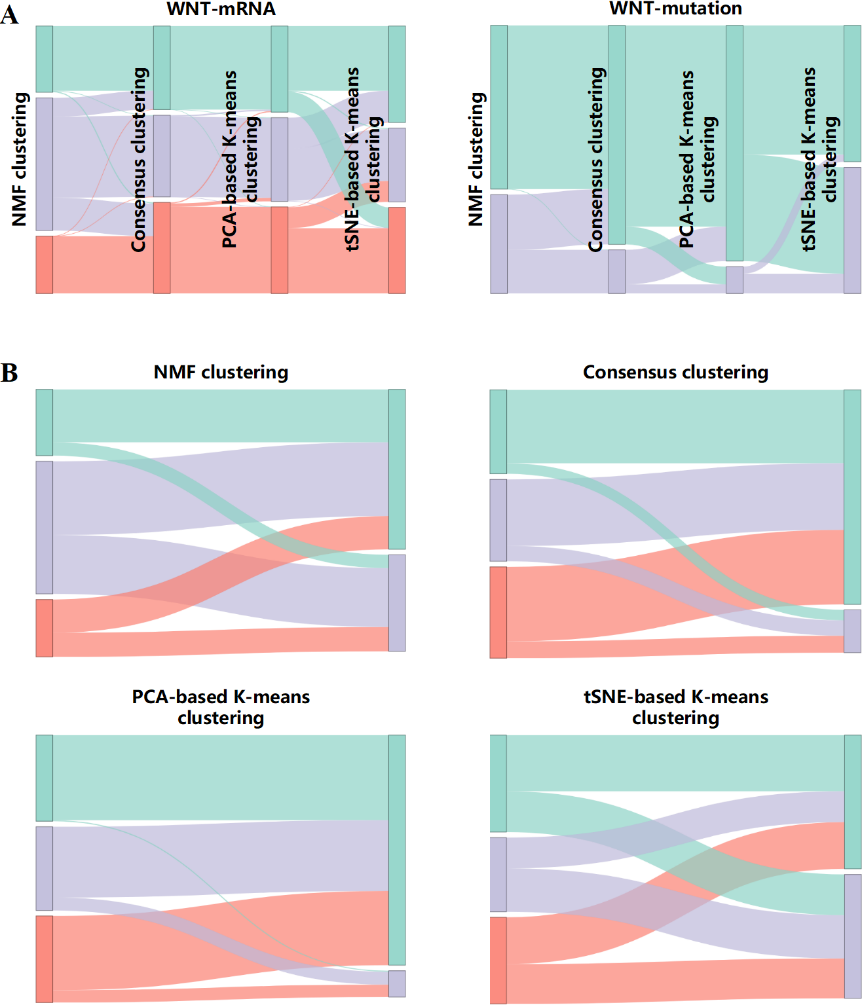


**Figure S3** The distribution of subtypes identified by consensus clustering, NMF clustering and K-means clustering with PCA or t-SNE analysis for WNT-mRNA and WNT-mutation data dimensionality reduction.


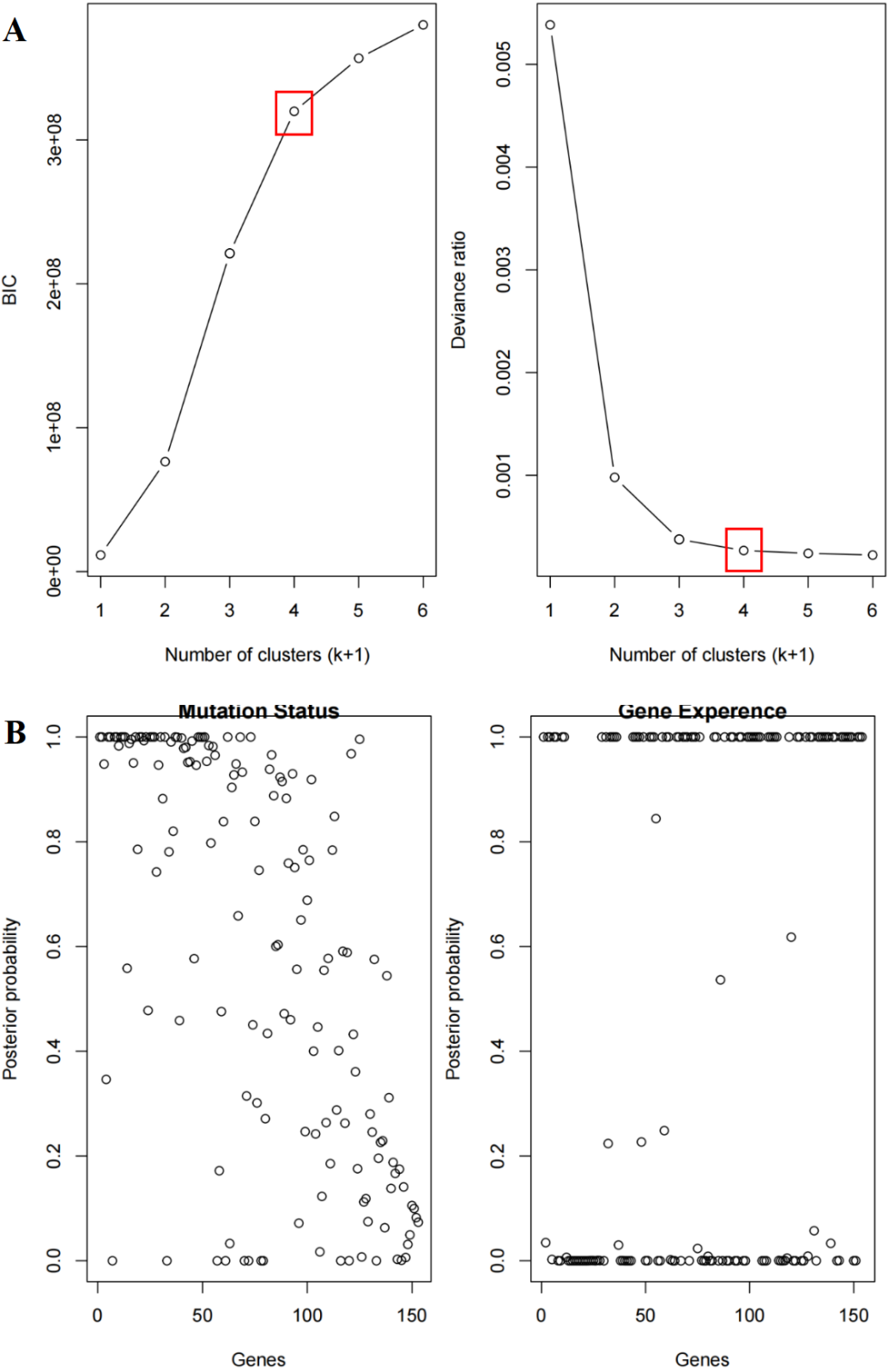


**Figure S4** Determination of the optimal number of clusters and classification characteristics for the comprehensive clustering of WNT pathway gene expression and mutation profiles. **(A)** Comprehensive clustering BIC index (left) and deviation ratio (right). **(B)** The prior probability of WNT pathway gene expression (left) and mutation status (right) in comprehensive clustering.


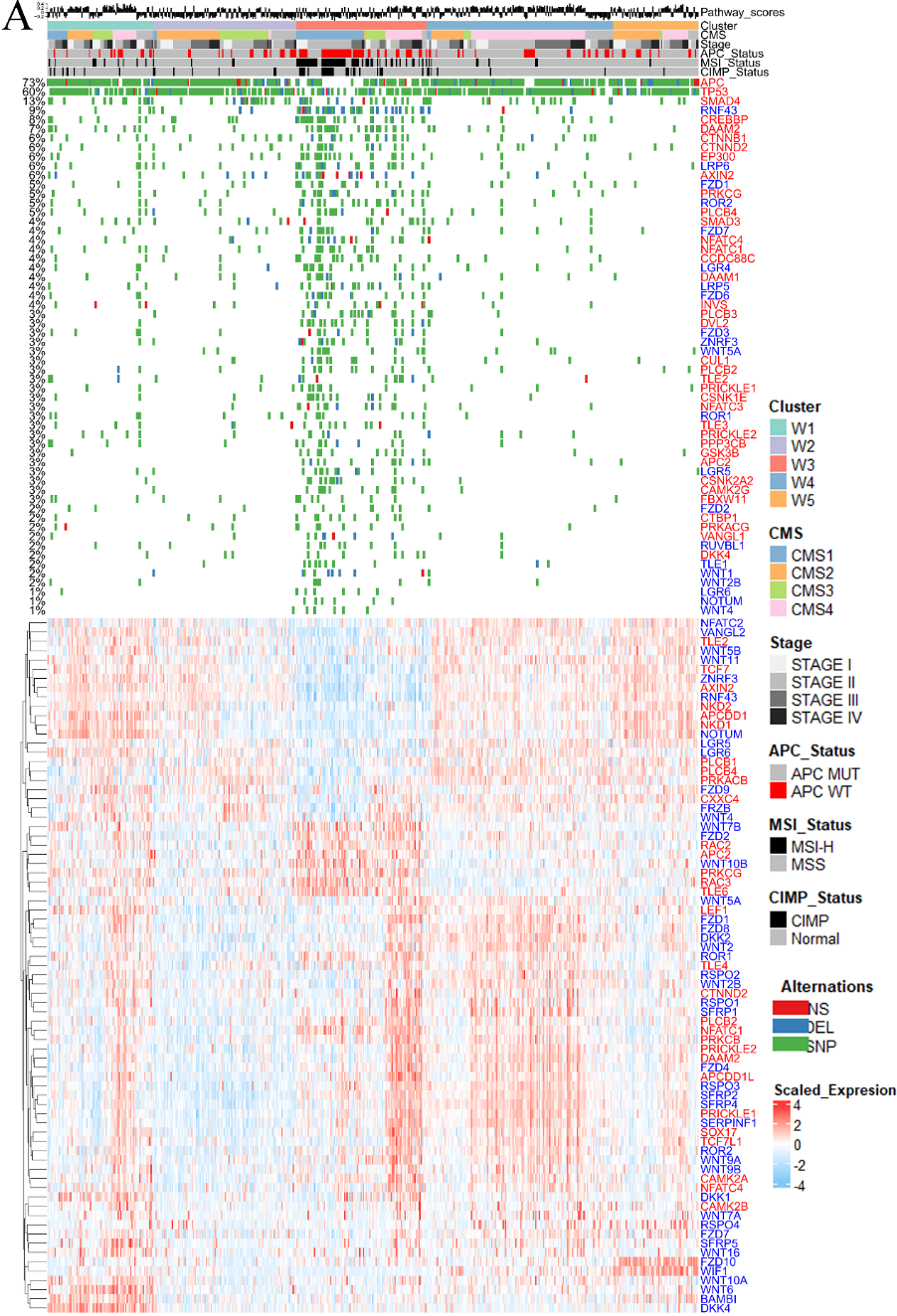

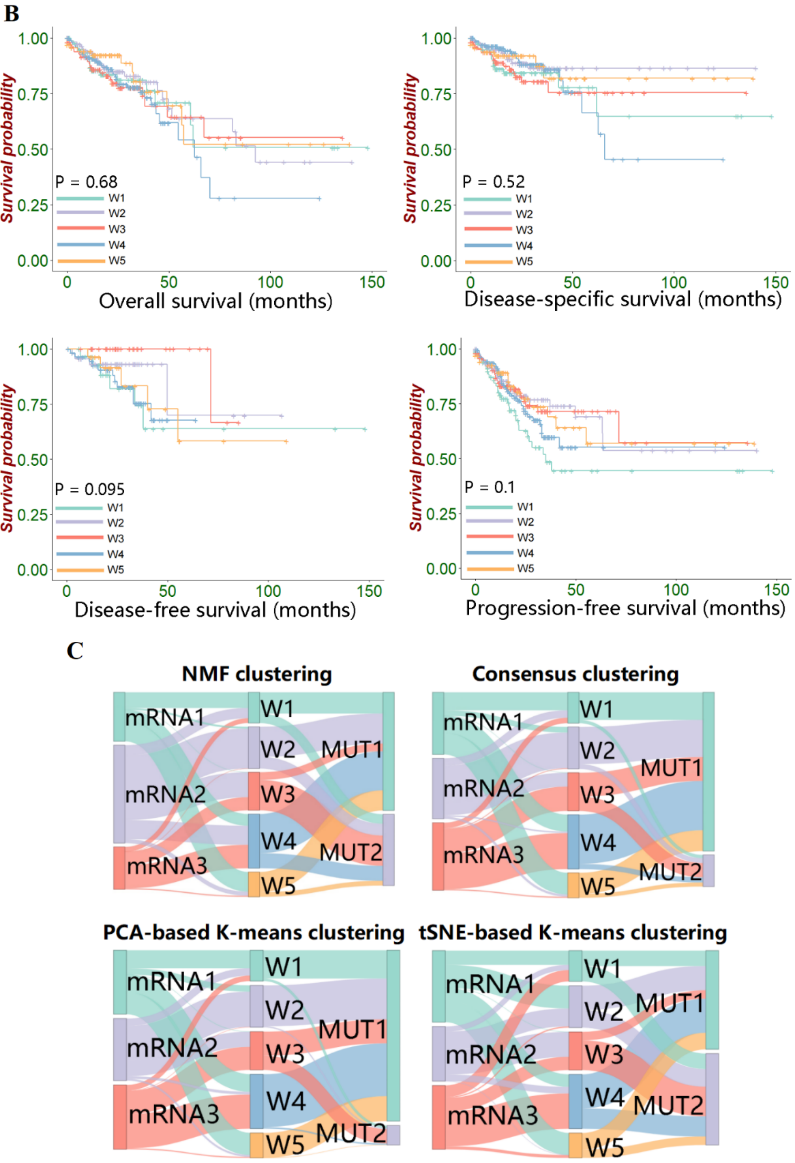


**Figure S5** Identification of CRC subtypes using WNT pathway gene expression profiles and mutation profiles. **(A)** Heatmaps display WNT pathway mRNA and mutation patterns of the CRC subtypes identified by comprehensive clustering (all features posterior probability > 0.9; blue gene names represent genes upstream of the WNT pathway and red gene names represent genes downstream of the WNT pathway). **(B)** Kaplan-Meier survival curves for the CRC subtypes of the TCGA dataset are presented for OS, DSS, PFS and DFS, respectively. **(C)** The distribution of subtypes identified by integrative clustering analysis for WNT pathway gene expression profiles and mutation profiles as well as consensus clustering, NMF clustering and K-means clustering with PCA or t-SNE dimensionality reduction for WNT pathway gene expression profiles and mutation profiles.


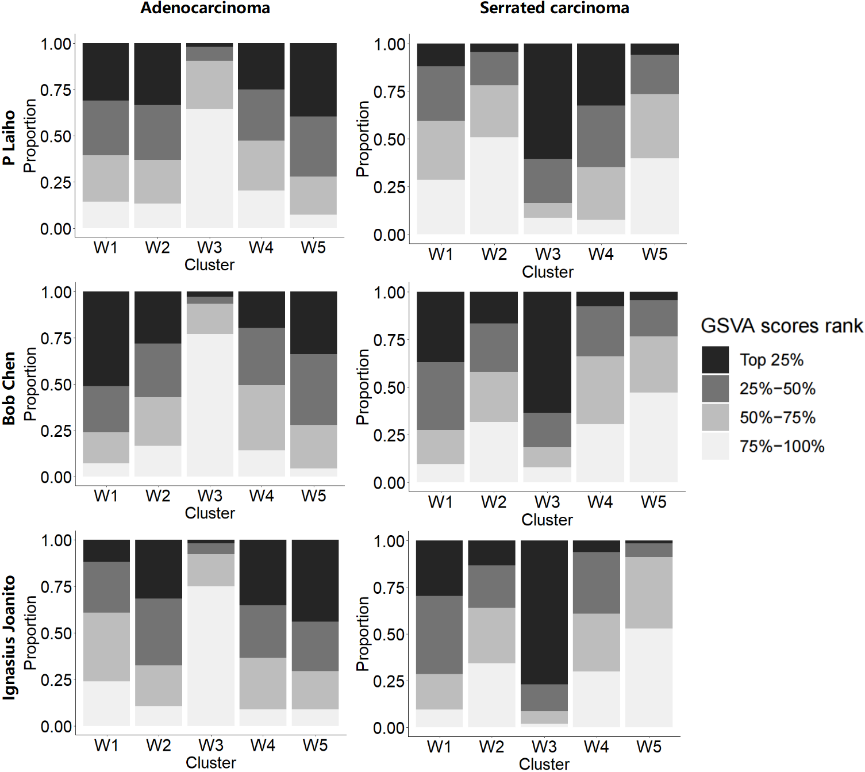


**Figure S6** GSVA ranking of serrated carcinoma and adenocarcinoma gene sets in other studies.


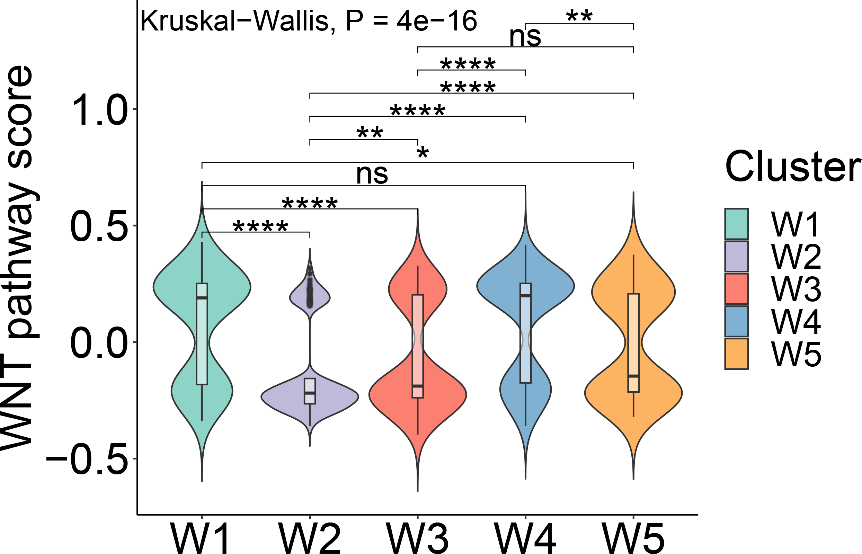


**Figure S7** WNT pathway activity of CRC subtypes in TCGA dataset.


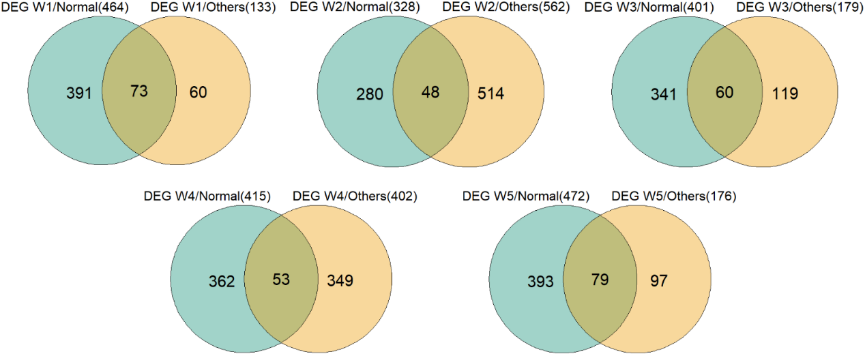


**Figure S8** The Venn plot shows the number of differentially upregulated genes for each subtype.


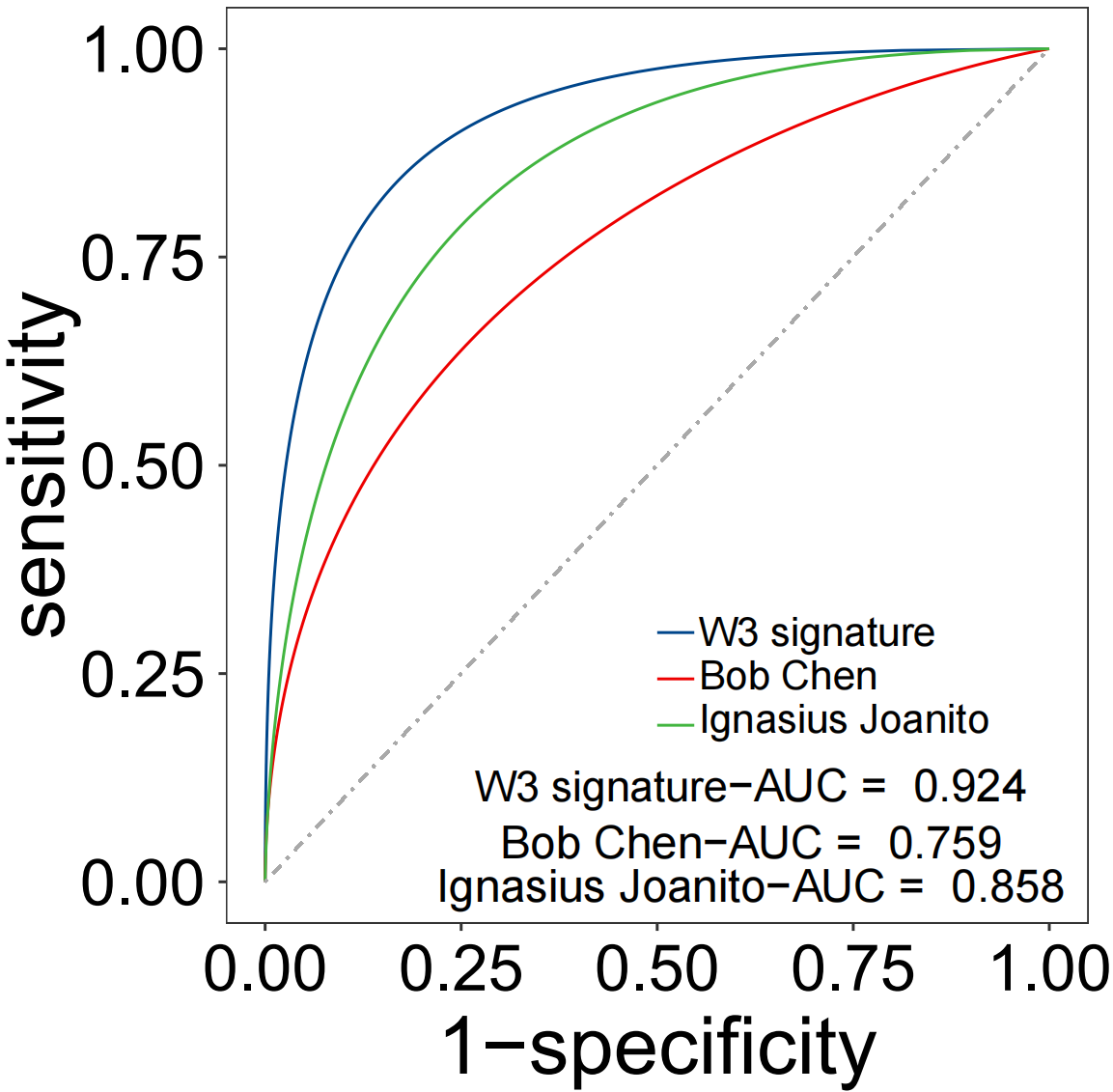


**Figure S9** ROC curves show the AUC values of the SCC signatures gene set and other serrated-related gene sets in TCGA training set.


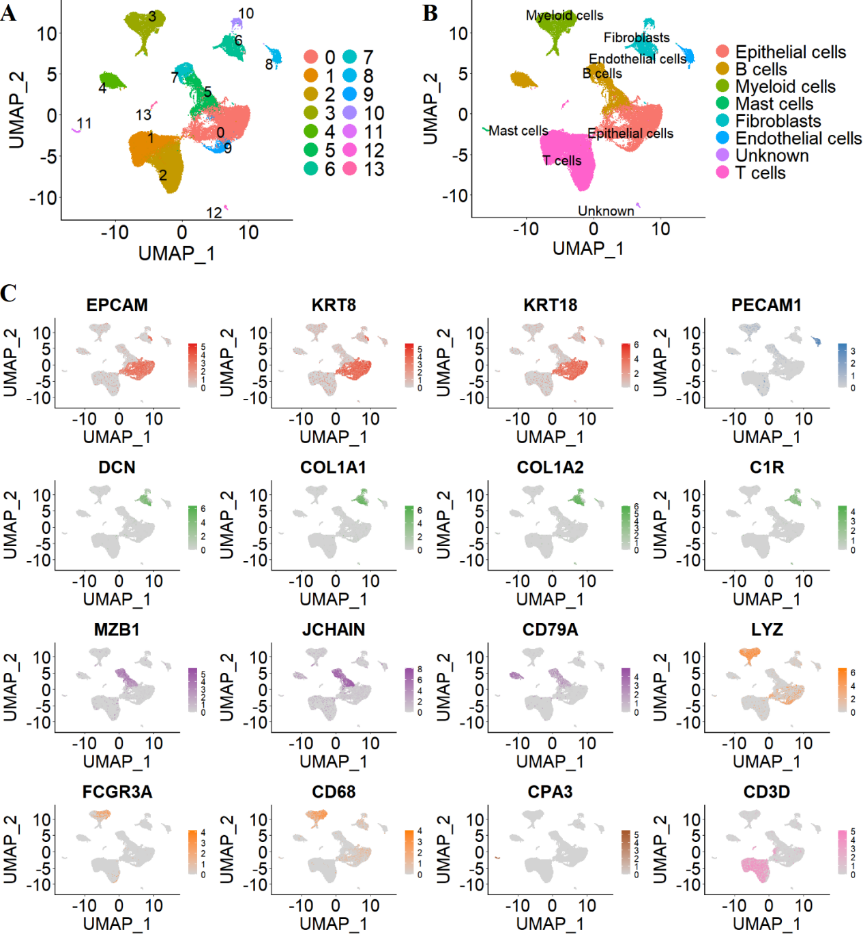


**Figure S10** Dissection of the various cell types in human CRCs with scRNA-seq. **(A)** UMAP plot show 14 clusters of 54,593 cells. **(B)** UMAP plot show the association of clusters with defined cell types. **(C)** Feature plots illustrate canonical markers of global cell types (epithelial cells: KRT18, KRT8 and EPCAM; endothelial cells: PECAM1; B cells: MZB1, JCHAIN and CD79A; T cells: CD3D; myeloid cells: LYZ, FCGR3A and CD68; fibroblasts: DCN, COL1A1, COL1A2 and C1R; mast cells: CPA3).


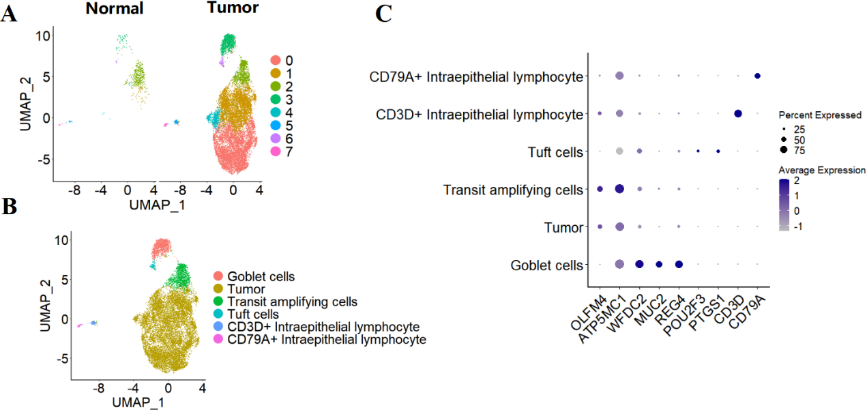


**Figure S11** Epithelial cell subclusters in CRC. **(A)** UMAP plot show the difference in distribution of the 8 clusters in normal versus tumor tissues. **(B)** UMAP plot show the association of clusters with defined types of epithelial cells. **(C)** Dotplot plots illustrate canonical markers of each normal epithelial cell (WFDC2, MUC2, and REG4), transit amplifying cells (OLFM4 and ATP5MC1), CD3D+ intraepithelial lymphocytes (CD3D), CD79A+ intraepithelial lymphocytes (CD79A), and tuft cells (POU2F3 and PTGS1).


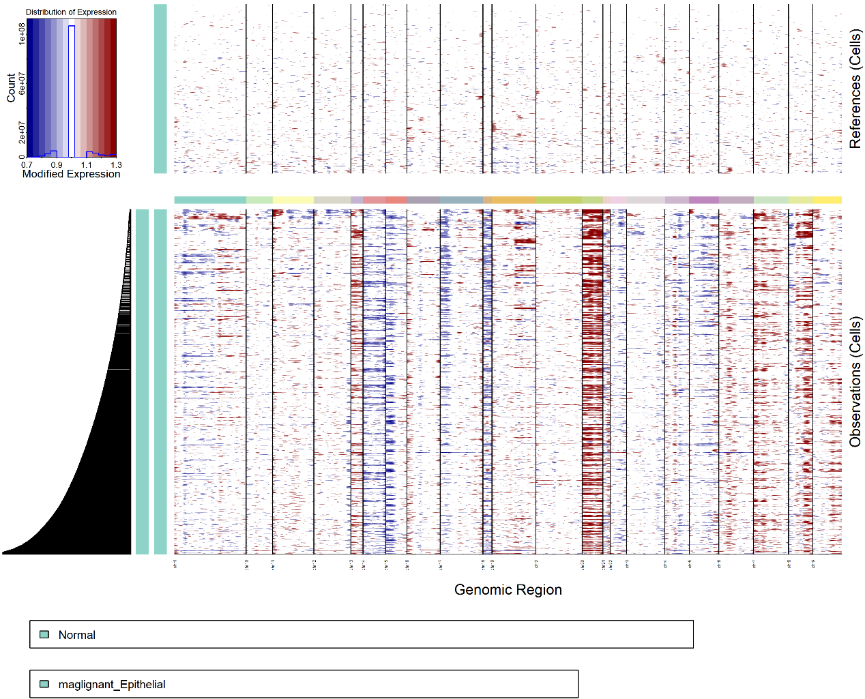


**Figure S12** The infraCNV heatmap displays the copy number variation of each colorectal cancer epithelial cell.


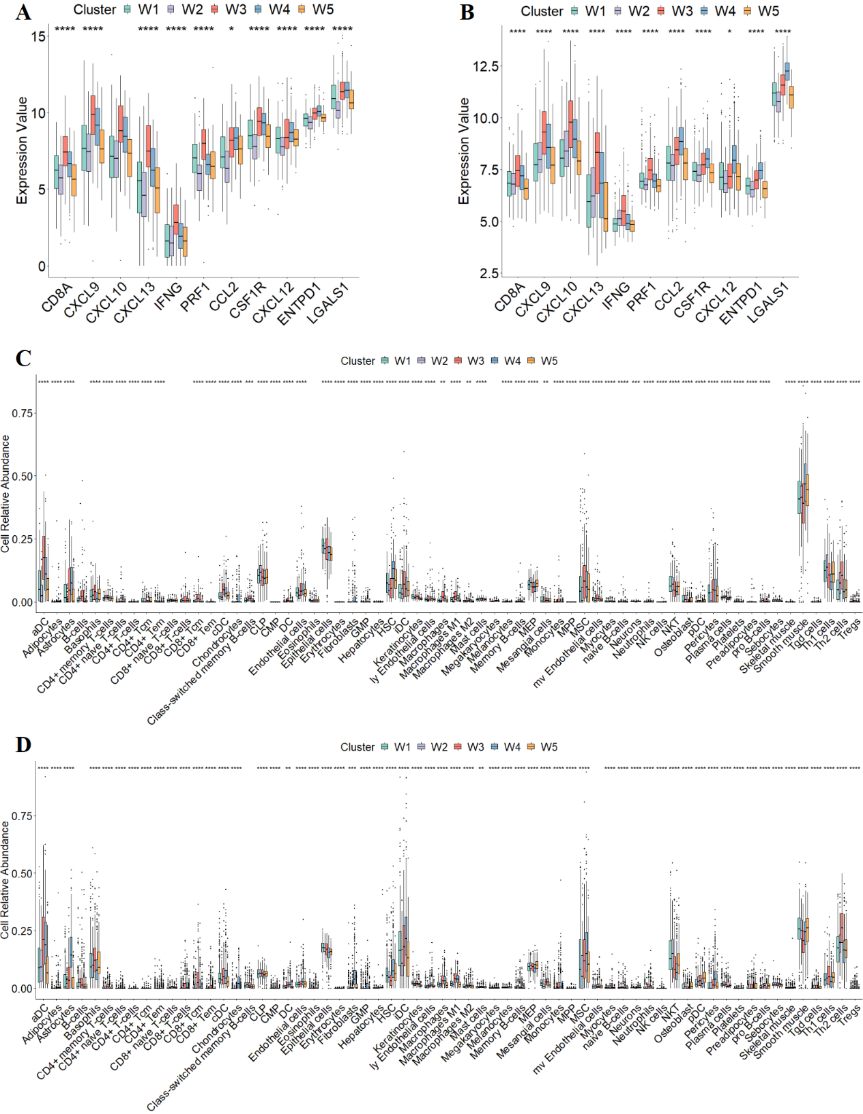


**Figure S13** Immune related genes and cell abundance of W1-W5 subtypes in TCGA (A and C) and GEO (B and D).
